# Editorial: Pulmonary immunity: role of inducible bronchus-associated lymphoid tissue in lung diseases

**DOI:** 10.3389/fimmu.2025.1725281

**Published:** 2025-10-29

**Authors:** Zhong-Min Ma, Rachel Reader

**Affiliations:** California National Primate Research Center, University of California, Davis, Davis, CA, United States

**Keywords:** pulmonary immunity, lung disease, bronchus associated lymphatic tissue (BALT), mucosa - associated lymphoid tissue, personal medicine, biological samples collecting

Lung diseases encompass a broad spectrum of disorders that continue to challenge clinicians and researchers alike. From chronic obstructive pulmonary disease to infections and malignancies, pulmonary diseases remain among the leading causes of death and disability worldwide. The COVID-19 pandemic and the rising incidence of early-age-onset lung cancer have only reinforced the urgency of understanding pulmonary immunity under both physiological and pathological conditions. At the center of this complex immune landscape lies the bronchus-associated lymphoid tissue (BALT)—a structure that is both a guardian of respiratory health and, paradoxically, a potential instigator of disease.

BALT was first described nearly half a century ago, building on histopathological observations of lymphoid aggregates within the lung made more than a century earlier. With the rise of mucosal immunology and the recognition of mucosa-associated lymphoid tissue (MALT), BALT came to be understood as a tertiary lymphoid structure within the bronchial wall. BALT is constitutively present in some species, such as rabbits and rats, but inducible in others, including humans and mice. This inducible form, often termed iBALT, develops in response to infection, inflammation, or environmental exposure. Despite its recognition, the biology of BALT—its structure, function, and role in disease—remains incompletely understood.

Recent investigations have revealed that the presence of BALT in the human lung is a context-dependent and developmentally regulated phenomenon. In infancy, BALT can develop naturally and function as a critical site for local immune priming. However, with aging, it tends to regress, re-emerging only upon immune challenge. This dynamic regulation redefines BALT as a physiological feature of mucosal immunity, one with potential implications for respiratory vaccination strategies and early-life immune education, rather than a pathological curiosity. In mouse models, virus-induced BALT has been shown to serve as a long-lasting priming site for T cell responses, dependent on dendritic cells for maintenance. These findings suggest that BALT may be a key structure for initiating and sustaining protective immunity in the lungs.

However, the story of BALT is not one of uniform benefit. Wein et al. reviewed that BALT has the potential to act as both a mediator of and a protector against graft rejection in lung transplantation. Early transplant studies in rats identified donor-derived BALT as a locus for graft rejection that facilitates local lymphocyte activation and antigen presentation. This interpretation was reinforced by observations in human lung allografts, in which BALT-like structures were associated with chronic rejection and inflammation. Consequently, BALT acquired a reputation as a pathological amplifier of immune injury.

However, a paradigm shift has emerged from more recent studies in murine models that more accurately reflect the inducible nature of human BALT. These studies have revealed that BALT is not invariably destructive; rather, its function is context-dependent. In tolerant lung allografts, for instance, BALT can serve as a sanctuary for immune regulation—rich in FoxP3^+^ regulatory T cells, dendritic cells, and specialized high endothelial venules. These microenvironments facilitate controlled immune cell trafficking and suppress effector responses, thereby maintaining both local and systemic tolerance. Indeed, clinical correlations now suggest that the presence of BALT in transplant recipients may coincide with lower rates of rejection and improved outcomes.

This dual nature—both protector and perpetrator—epitomizes the complexity of pulmonary immune regulation. Whether BALT contributes to protection or pathology appears to depend on multiple variables: the species in question, the nature of antigen exposure, the local cytokine milieu, and the balance between effector and regulatory immune populations. In conditions such as chronic infection, autoimmune disorders, or COPD, inducible BALT may perpetuate inflammation. Conversely, in controlled immune contexts such as transplant tolerance or respiratory vaccination, it may sustain protective immunity.

As our understanding deepens, the need for precision in defining and characterizing BALT becomes increasingly apparent. The term itself has been applied to diverse lymphoid aggregates throughout the lung, with varying criteria for inclusion. Some define BALT strictly as organized lymphoid structures containing B cell follicles and follicular dendritic cells; others use the term more broadly to encompass any peribronchial or perivascular lymphoid cluster. Mikami et al. reported that lymphoid tissue was found around blood vessels in mice after exposure to Asian sand dust (ASD). Ma et al. proposed the term pulmonary lymphoid tissue (PLT) to better capture the diversity and distribution of these immune aggregates across the lung parenchyma—a terminology shift that could unify research efforts under a more inclusive framework.

Future progress will depend on technological advances that enable precise monitoring of pulmonary immunity. Modern imaging, transcriptomics, and biomarker analyses—ranging from tissue and fluid samples to exhaled breath—offer unprecedented tools with which to characterize BALT and related structures in situ. For instance, transcriptomic profiling of alveolar macrophages, as demonstrated by Du et al. in viral infection models, provides a template for dissecting the molecular networks that govern pulmonary lymphoid organization. A standardized framework for assessing the “status” of BALT or PLT could revolutionize how clinicians interpret immune landscapes in the lungs and guide individualized interventions.

Harnessing the beneficial aspects of BALT represents an exciting frontier for translational medicine. Strategies that promote the formation of regulatory BALT—through cytokine modulation (e.g., IL-22), adoptive Treg therapy, or controlled antigen exposure—could enhance mucosal protection while minimizing inflammation. These approaches may not only improve outcomes in lung transplantation but also pave the way for novel respiratory vaccines and immunotherapies that target chronic lung diseases.

In summary, the history of BALT research mirrors the evolution of modern immunology itself: from morphological observation to molecular dissection and from pathology to precision medicine. What was once regarded as a structure of rejection may, under the right conditions, become a cornerstone of pulmonary tolerance and defense. The challenge now is not to suppress or eliminate BALT, but to understand and shape it—to turn this once-controversial lymphoid aggregate into a clinical ally in the fight against lung disease.

